# Evolutionary honing in and mutational replacement: how long-term directed mutational responses to specific environmental pressures are possible

**DOI:** 10.1007/s12064-023-00387-z

**Published:** 2023-03-11

**Authors:** Adi Livnat, Daniel Melamed

**Affiliations:** 1 Department of Evolutionary and Environmental Biology, University of Haifa, 3498838 Haifa, Israel; 2 Institute of Evolution, University of Haifa, 3498838 Haifa, Israel

**Keywords:** Interaction-based evolution, Fusion mutation, Parallelism, Genome organization, Nonrandom mutation, Hemoglobin S

## Abstract

Recent results have shown that the human malaria-resistant hemoglobin S mutation originates de novo more frequently in the gene and in the population where it is of adaptive significance, namely, in the hemoglobin subunit beta gene compared to the nonresistant but otherwise identical 20A$\rightarrow$T mutation in the hemoglobin subunit delta gene, and in sub-Saharan Africans, who have been subject to intense malarial pressure for many generations, compared to northern Europeans, who have not. This finding raises a fundamental challenge to the traditional notion of accidental mutation. Here, we address this finding with the replacement hypothesis, according to which preexisting genetic interactions can lead directly and mechanistically to mutations that simplify and replace them. Thus, an evolutionary process under selection can gradually hone in on interactions of importance for the currently evolving adaptations, from which large-effect mutations follow that are relevant to these adaptations. We exemplify this hypothesis using multiple types of mutation, including gene fusion mutations, gene duplication mutations, A$\rightarrow$G mutations in RNA-edited sites and transcription-associated mutations, and place it in the broader context of a system-level view of mutation origination called interaction-based evolution. Potential consequences include that similarity of mutation pressures may contribute to parallel evolution in genetically related species, that the evolution of genome organization may be driven by mutational mechanisms, that transposable element movements may also be explained by replacement, and that long-term directed mutational responses to specific environmental pressures are possible. Such mutational phenomena need to be further tested by future studies in natural and artificial settings.

## Introduction

Mutation rates have long been measured as averages across many genomic positions: across the whole genome, across instances of a motif, across the stretch of a gene, etc. (Kondrashov 2003; Hodgkinson and Eyre-Walker 2011; Rahbari et al. 2016; Carlson et al. 2018). From these measurements, it has already been known that mutation rates vary on multiple scales—from the chromosomal scale to differences between motifs (Hodgkinson and Eyre-Walker 2011; Rahbari et al. 2016; Carlson et al. 2018). However, it has not been widely considered that this nonuniformity could carry meaningful biological information that is of essence to the fundamental principles of how evolution happens, and this possibility has not been tested until recently at the resolution of specific mutations as opposed to mutation rate averages.

Recently, we developed a method to measure mutation rates at the single-mutation resolution and applied it to a 6 bp region in the human hemoglobin subunit beta (*HBB*) gene that contains the site of the malaria-resistant HbS mutation and to the identical region in the hemoglobin subunit delta (*HBD*) gene, in sperm samples from both sub-Saharan African and northern European donors (Melamed et al. 2022). Results showed that mutation rates at the single-mutation resolution varied much more and in a different manner than expected from previous studies based on average mutation rates (cf. Harris 2015; Harris and Pritchard 2017). For example, mutation-specific rates varied substantially between the two paralogs and between the two populations, even though the mutations appeared on the identical local genetic sequence (Melamed et al. 2022), suggesting that much of the signal in mutation rate variation could be in mutation-specific rates and thus had not been measurable before. Furthermore, the overall point mutation rate in *HBB* was significantly higher in Africans than in Europeans (Melamed et al. 2022), a difference at least two orders of magnitude larger than previously measured differences between human populations (Harris 2015; Harris and Pritchard 2017). Finally, a combination of statistically significant tests showed that the HbS mutation originates de novo more frequently in the *HBB* gene, where it provides resistance to malaria, compared to the nonresistant but otherwise identical mutation in *HBD*, and in sub-Saharan Africans, who have been experiencing intense malarial selection pressure for many generations, compared to northern Europeans, who have not (Melamed et al. 2022). Thus, it originates de novo more frequently where it is of adaptive significance.

To explain mutation rate patterns in accord with the traditional view, one often invokes modifier theory (Leigh Jr 1970; Feldman and Liberman 1986; Altenberg and Feldman 1987; Altenberg et al. 2017). According to this theory, a random mutation that changes the average mutation rate can be fixed by natural selection, provided that it changes the average mutation rate across a large enough number of loci with which it remains linked for a long enough period of time; in this manner, many potential mutations, each occurring with only a small probability, can figure into its selective advantage (Hodgkinson and Eyre-Walker 2011; Martincorena and Luscombe 2013; Walsh and Lynch 2018; Monroe et al. 2022). For example, if a random mutation in a DNA repair factor causes the latter to repair DNA with higher accuracy, this mutation could be favored by selection. Accordingly, it was recently argued that the evolution of mutation rate modifiers reduced the average mutation rate in *Arabidopsis thaliana* in essential genes, which constitute ${\sim 30\%}$ of the plant’s genome according to the authors (Monroe et al. 2022). However, because modifier theory requires the modifier allele to affect a sufficiently large number of loci, it cannot explain increases in the rates of specific mutations at specific base positions (Leigh Jr 1970; Feldman and Liberman 1986; Hodgkinson and Eyre-Walker 2011; Altenberg et al. 2017; Martincorena and Luscombe 2013; Walsh and Lynch 2018), and thus it cannot explain the patterns observed in the hemoglobin study. Furthermore, since *HBB* is an essential gene, only low mutation rates across it could have been predicted based on the claim that essential genes are better protected from mutations (Monroe et al. 2022).

Given all of the above, how can the hemoglobin findings be interpreted? One possibility is that humans have a genomic fragility that, by coincidence, causes rapid origination of the malaria-resistant HbS mutation both in the gene and in the population where it is of adaptive significance. However, this would leave anomalous data from the first measurement of mutation rates at the single-mutation resolution, regarding a flagship example of adaptation by random mutation and natural selection (Freeman and Herron 1998; Hartl and Clark 2007).

Another hypothetical possibility is that the HbS mutation originates as a Lamarckian, direct response to the immediate environment (Luria and Delbrück 1943; Cairns et al. 1988; Sarkar 1990). However, given the fundamental limitations of Lamarckism (e.g., Haig 2007), this possibility will not be considered here.

A third possibility, which we will focus on here, is that the HbS mutation originates in a manner that is neither accidental nor Lamarckian. According to this possibility, this mutation demonstrates a long-term directed mutational response to a specific environmental pressure (Livnat 2013, 2017). But what does long-term directedness mean, and how could the genome “know” to generate a mutation more frequently where it is of adaptive significance? Our goal in this paper will be to propose an answer in outline that is not susceptible to the conceptual difficulties of purely random mutation, modifier theory or Lamarckism.

## The used-fused hypothesis for nonrandom gene fusion mutations

We begin with an example that may at first seem unrelated, but later will be seen as directly relevant. Consider the TRIM5 and cyclophilin A encoding genes, which fused at least twice independently by retroposition in at least two different simian lineages (Virgen et al. 2008; Nisole et al. 2004; Sayah et al. 2004; Liao et al. 2007; Brennan et al. 2008; Wilson et al. 2008; Newman et al. 2008). This recurrent fusion produced a fusion protein (TRIM5-CypA) that protects against certain lentiviruses, including HIV-1 (Nisole et al. 2004; Sayah et al. 2004). It is difficult to attribute the independent recurrence of such a gene fusion mutation to chance. Whereas in the case of a parallel point mutation, a single base position has to repeat independently, in the case of recurrent gene fusion, multiple similar breakpoints must repeat independently. Mathematically, if the probability of the former is small, the probability of the latter is negligible.

However, genes that are used together are more likely to be transcribed at the same time and place in the nucleus—for example in co-expression domains (Soler-Oliva et al. 2017), topologically associating domains (Dixon et al. 2012) and transcription factories (Jackson et al. 1993; Edelman and Fraser 2012; Papantonis and Cook 2013) where DNA loops bring also remote coactive genes together. Therefore, to explain gene fusion, we argued that the presence of the two coactive genes at the same time and place, with the chromatin open at both loci due to transcription, enables various downstream mechanisms, such as reverse transcription of the RNA (perhaps aided by trans-splicing), transposable element–mediated translocation, recombination-based mechanisms, DNA breaks induced by the spatial chromosomal organization and active gene transcription, and other mechanisms to generate a gene fusion (Livnat 2017; Bolotin et al. 2022). We furthermore hypothesized that because the genetic information that indicates that the two genes work together, such as shared cis elements and transcription factors that bind to them, is present in the DNA and accessible in the germline, this fusion effect applies not only to pairs of genes that serve germline functions but also to pairs that serve somatic functions (Livnat 2017; Bolotin et al. 2022). Indeed, the germline-specific phenomenon of transcriptional promiscuity allows many somatic genes to be regularly transcribed in the germline (Kleene 2005; Melé et al. 2015; Xia et al. 2020) and thus to participate in mutational mechanisms involving interactions between genes (Livnat 2013, 2017). Thus, we hypothesized that genes that are used together repeatedly and persistently in a certain context are incomparably more likely than other genes to be fused together by a mutational mechanism (Livnat 2017; Bolotin et al. 2022). In other words, it is genes that are used together that can be fused together in the course of evolution—henceforth the “used-fused” effect. This hypothesis offered the first explanation for why there are recurrent gene fusions, and why they are common, both in evolution (Carvalho et al. 2010; Livnat 2013) and in genetic disease and cancer (Li et al. 2008; Osborne 2014). Recently, we found empirical support for this hypothesis, including its applicability to both germline and somatic genes (Bolotin et al. 2022).

This used-fused effect has multiple consequences. First, it reverses the two key assumptions of modifier theory. While in modifier theory, the average mutation rate across many loci is presumed to be affected in a blanket manner (Walsh and Lynch 2018), here, the used-fused effect increases the fusion probability specifically of the two genes that interact. Furthermore, while in modifier theory the mutation rate across many loci can be attributed to a single modifier allele (Walsh and Lynch 2018), here, the genetic information that causes the increase in the rate of a particular fusion mutation is complex: it involves all of the information that makes these two genes interact tightly, such as promoters, enhancers, transcription factors and epigenetic marks of the two interacting loci and others that regulate them.

Second, the outcome of the fusion can be seen as local mutational simplification of gene regulation (Livnat 2017; Bolotin et al. 2022): before the fusion, the two genes had to be activated separately and their products had to meet, whereas the fusion has chunked them together into a single unit that can be activated as one.

Third, this effect demonstrates that a gradual, long-term process of evolution of regulation can pave the way to a discrete mutational change (Livnat 2013, 2017; Bolotin et al. 2022; Melamed et al. 2022). It may have seemed as though a gene fusion mutation must arise by a sudden evolutionary jump, where a sequence is randomly translocated from one context to another. However, according to the used-fused hypothesis, the genetic interaction that leads to a fusion mutation has already evolved and repeatedly occurred through the generations. In other words, two genes first come to interact with each other tightly in the course of evolution through the gradual accumulation of multiple other heritable changes of smaller effect in various loci, which in turn leads to a situation where the mutation that fuses them becomes more likely to arise (Livnat 2017; Bolotin et al. 2022).

Fourth, it demonstrates that local simplification can lead to a global increase in complexity (Livnat 2017). Although the used-fused effect leads to local simplification, it does not lead to ever more simplicity and diminution of the genome; quite the contrary: fusion often comes together with or is preceded by gene duplication of the source copies and therefore often leads not from two genes to one, but from two genes to three. This increases the genetic vocabulary and the extent of interactions between genes, and thus, in the long term, it increases complexity (Livnat 2017; Bolotin et al. 2022).

Fifth, it demonstrates the evolution of innateness at the molecular level. Because the two genes previously had to be brought together in order to interact, and now they are genetically fused and activated as one, a phenotype—an interaction between genes—has been replaced with an innate, ready-made object—a gene (Livnat 2017). This endogenization by mutation brings the causes of mutation to bear on genetic assimilation (contrast with Waddington 1953; West-Eberhard 2003; Laland et al. 2015), as will be discussed later.

## The replacement hypothesis is an extension of the used-fused hypothesis

The used-fused effect is one of multiple examples of a broader hypothesis, called “the replacement hypothesis,” that an evolved, system-level phenomenon can lead directly and mechanistically to a new and specific mutation that replaces and simplifies this pre-evolved phenomenon (Melamed et al. 2022). We elaborate on this hypothesis below through examples.

### Gene duplication as mutational replacement

The mutational phenomena that enable whole gene duplication (e.g., non-allelic homologous recombination; Gu et al. 2008) are essential for long-term evolution. They can generate two functional gene copies from one, thus enabling the copies to specialize by the gradual accumulation of mutations under selection (Ohno 1970). Here, we hypothesize that these mutational phenomena could be even more useful in the long term than previously considered.

Several findings show that elevated transcription increases the chance of a gene duplication mutation (Hull et al. 2017; Wilson et al. 2015; Thomas and Rothstein 1989; Aguilera and Gaillard 2014; Hamperl et al. 2017), likely by local destabilization of the genome (Aguilera 2002; Gómez-González and Aguilera 2019) followed by downstream mechanisms involving RNA (Kaessmann et al. 2009) or DNA intermediates (Chicote et al. 2020; Durkin et al. 2012; Takahashi and Innan 2020). A direct connection between transcription and gene duplication mutation rates was exemplified in yeast, where copy number amplification of the CUP1 gene results in adaptation to high copper concentration: by replacing the CUP1 promoter sequence with a galactose-inducible promoter, Hull et al. (2017) have shown that CUP1 transcription induces gene copy number amplification under galactose stimulation and in the absence of copper selection, suggesting that copy number amplification of the wild-type CUP1 can be caused mechanistically by transcription. The mechanism likely involves transcription-inducible formation, accumulation and reintegration of extrachromosomal circular DNA (eccDNA) (Hull et al. 2017).

How shall we interpret this mechanistic link between elevated transcription and gene duplication mutation? Examining it from the random mutation perspective, it is merely one phenomenon untied to others. Examining it from a Lamarckian perspective, it is limited in its ability, as it can facilitate evolutionary adaptation mainly in unicellular organisms in certain situations. However, Livnat (2017) has argued that heritable change in general is neither accidental nor Lamarckian: instead of only responding directly to an environmental cue, regulatory activity that has gradually evolved through the generations and has come to cause elevated transcription can increase the probability of a gene duplication mutation in the germline. Then, the gene duplication mutation replaces the previous regulatory activity with an innate ability to produce a large amount of the gene product (Livnat 2017).

As in the case of the used-fused mechanism, Livnat (2017) has argued that this elevated transcription–based gene duplication mechanism applies not only to genes that serve germline functions but also to genes that serve somatic functions, due to germline expression of somatic genes, without a Lamarckian transmission of information from the soma to the germline. Evidence exists consistent with the operation of the focal mechanism in sperm (Vinckenbosch et al. 2006; Park et al. 2012; Mouakkad-Montoya et al. 2021). Also as in the case of gene fusion, this mechanism may facilitate long-term evolution: where high quantities of the gene product are needed, it could alleviate the pressure on gene expression, and where usage of a gene by multiple processes leads to its high expression, duplication and specialization of it into two or more paralogs could facilitate evolution by resolving pleiotropy (Livnat 2017).

Because this gene duplication mechanism replaces preexisting regulatory activity with an innate ability to produce a large amount of the product, it is an example of the replacement hypothesis; its action in general is not an immediate and direct response to the environment, and it facilitates the evolution not only of unicellular but also of multicellular organisms.

### Mutational replacement of RNA editing

Following the common posttranscriptional regulatory mechanism carried out by members of the adenosine deaminase acting on RNA family (ADARs), called A-to-I RNA editing, the inosine (I) is usually recognized as guanine (G) during mRNA translation (Bass 2002). Comparing RNA A-to-I edited sites to non-edited sites in one species, the former are more likely to correspond to sites in other species where A$\rightarrow$G DNA substitutions occurred (Grauso et al. 2002; Ohlson et al. 2007; Tian et al. 2008; Xu and Zhang 2014; Chen et al. 2014), and are also more likely to exhibit A/G DNA polymorphisms in the same species (Popitsch et al. 2020; Danecek et al. 2012). In *Drosophila melanogaster*, for example, A/G DNA polymorphisms are approximately twice more common in A-to-I edited than non-edited sites, and the polymorphisms almost always (98%) feature G rather than C or T (Popitsch et al. 2020). Interpreting these data from the random mutation perspective, some authors suggested that A-to-I editing is a rescuing mechanism from past, deleterious G$\rightarrow$A substitutions (Pinto et al. 2014); others suggested that both A-to-I RNA editing and A$\rightarrow$G DNA mutations largely represent promiscuous, erroneous activity that is only slightly deleterious, and therefore in sites where nonfunctional A-to-I RNA editing is tolerated by selection, random A$\rightarrow$G mutations are also more likely to be tolerated by selection (Xu and Zhang 2014); and yet others suggested that selection favors G in the RNA-edited sites, and therefore random A$\rightarrow$G mutations will be favored by selection there too (Popitsch et al. 2020). Thus, different groups of authors have been led to contradictory conclusions.

In contrast, we have argued that the evolutionary increase through the generations in the rate of RNA editing directly and mechanistically generates an increase in the rate of the corresponding DNA mutation in the corresponding positions (Melamed et al. 2022). Indeed, evidence has accumulated suggesting a mechanistic connection between RNA editing and DNA mutation either by ADAR acting directly to mutate transcribed DNA (Shiromoto et al. 2021; Jimeno et al. 2021; Zheng et al. 2017; Tsuruoka et al. 2013) or via reverse transcriptase activity of several DNA polymerases (Chandramouly et al. 2021; Su et al. 2019; Franklin et al. 2004) using edited RNA as a template.^1^

**Fig. 1:**
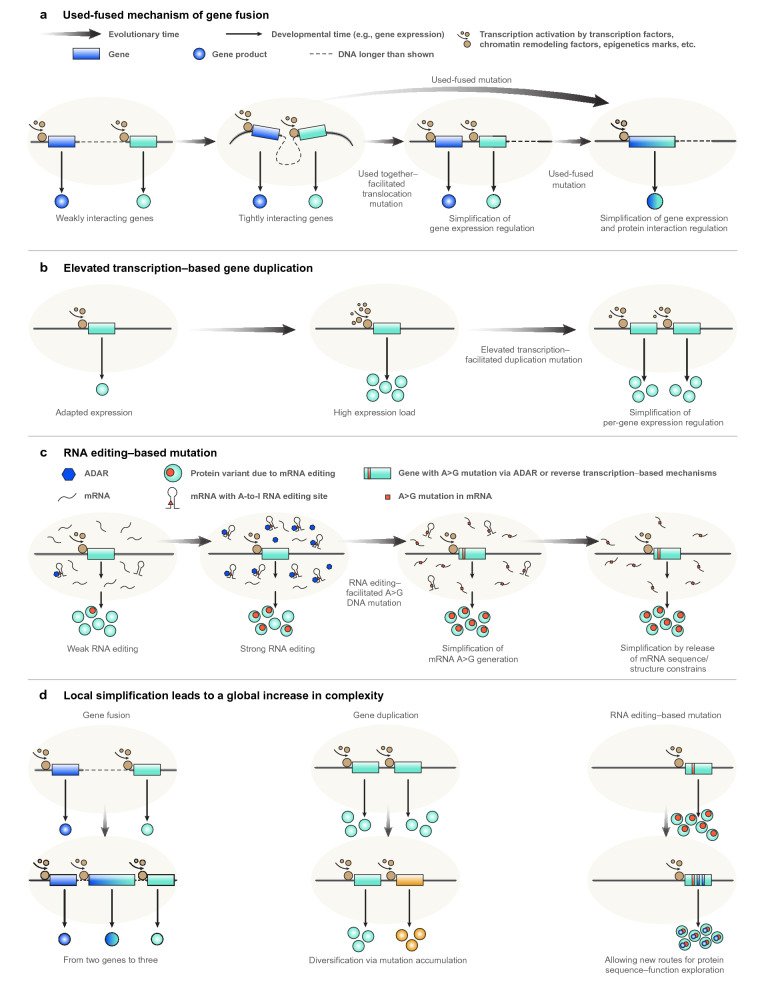
Gene fusion, gene duplication and RNA editing–based mutations as examples of the replacement hypothesis. **a** The gradual evolution of tight interaction between two genes precedes their translocation or fusion. This interaction brings together the two loci with their chromatin open at the same time and place in the nucleus in co-expression domains, topologically associating domains or transcription factories, thus enabling a translocation mutation that makes them neighbors or a fusion mutation that makes them into one gene by various downstream mutational mechanisms. The translocation or fusion mutations obviate preexisting regulatory activity that was needed to bring the two genes together, thus replacing it with a simplified, hardwired, innate state. **b** Increased expression beyond the norm for a certain gene makes it more likely to undergo a duplication mutation. For CUP1 in yeast, the generation of eccDNA and its reintegration into the genome was implicated. The duplication mutation obviates the need for excessive transcription through regulation, replacing it with a locally simplified, hardwired, innate state. **c** The evolution of A-to-G RNA editing of a certain site comes together with an increase in the rate of an A$\rightarrow$G mutation at the same position in the DNA. This mutation obviates the need for the RNA editing regulatory activity, thus replacing it with a simplified, hardwired, innate state. **d** According to the theory of interaction-based evolution (IBE), the simplification of interactions generates elements that engage in new interactions with other such elements at the system level. Thus, in the context of gene duplication, local simplification leads to a global increase in complexity: the fusion of genes leads from two genes to three, thus increasing the number of genes, the overall extent of interactions between genes, and complexity (left); in the long term, gene duplication enables the two copies to undergo differential accumulation of mutations, leading to specialization and complexity (center); and the accumulation of point mutations is a part of the evolution of complexity (right)

The fact that the correspondence between A-to-I RNA editing on the one hand and A$\rightarrow$G DNA substitutions as well as A/G DNA polymorphisms on the other has been observed for both coding and noncoding regions as well as for both synonymous and nonsynonymous A$\rightarrow$G changes (Popitsch et al. 2020) is difficult to explain by random mutation and natural selection (rm/ns^2^). In contrast, we argue that the following of A-to-I RNA editing in evolutionary time by A$\rightarrow$G mutations in the corresponding DNA positions is not due to rm/ns but is another example of the mutational replacement hypothesis. Following such replacement, regulation at the phenotypic level is no longer needed to obtain the A-to-G outcome, and thus the replacement can be seen as a form of local simplification. Thus, gene fusion, gene duplication, A$\rightarrow$G mutations in RNA-edited sites and other mutations to be mentioned later all fit under a unifying umbrella, that of the replacement hypothesis (Fig. 1).

## A system-level view of mutation origination

What is “random mutation”? According to one definition, “random mutation” means that the ultimate causes of mutation are not meaningfully related to their biological consequences or to the structure and function of the organism—they are accidental (physicochemical damage to the molecular structure of the DNA, thermal fluctuations during DNA replication, etc.) (Freeman and Herron 1998), and thus their detail is irrelevant for the core principles of how evolution happens. According to another definition, random mutation is defined in contrast to Lamarckism—it is a mutation that is not Lamarckian (Futuyma 1998)—where Lamarckism means that the organism responds directly to the immediate environment by generating beneficial heritable change that alleviates the environmental pressure. Implicitly or explicitly, these definitions have been used interchangeably, implying that mutation is either fundamentally accidental or Lamarckian (Morgan 1903; Fisher 1930; Dawkins 1986; Lenski and Mittler 1993; Futuyma 1998; Merlin 2010; Razeto-Barry and Vecchi 2017).

According to the theory of interaction-based evolution (IBE) (Livnat 2013, 2017), the heritable changes that drive adaptive evolution under selection are neither accidental nor Lamarckian. They are not accidental, because the probability of origination of each heritable change depends on complex information in the genome (i.e., genetic interactions) in a biologically meaningful manner. They are not Lamarckian, because they are influenced not by information that arrives directly from the immediate environment but by information that has accumulated in the germline genome through the generations. This information, in turn, has come from previous heritable changes and previous selection pressures (Livnat 2013, 2017). Thus, evolution is driven by the interaction of two forces: the external force of differential survival and reproduction and the internal force of nonrandom, non-Lamarckian heritable change, both of which are continually updated: as the organism gradually changes through the generations, so does the selection pressure change, because it depends on the organism and how it fits with its environment; and as the genome gradually changes, so do the mutation-specific probabilities of mutation origination change, because they are influenced by the genome in a complex manner. Both forces continually interact as they coevolve, and their interaction drives evolution (Livnat 2013, 2017).

According to this theory, the distribution of mutation rates across the genome carries meaningful biological information at the mutation-specific level and is continually evolving. Therefore, its particular form at any one time is critical for the evolution of adaptations occurring at that time. This theory differs from modifier theory in arguing that mutation rates are mutation-specific and determined by complex information in the genome, and that the fundamental nature of mutation origination is not accidental to begin with. It implies that selection does not act on mutation rates as captured by models of modifier theory; rather, it acts on the complex phenotype, changing allele frequencies and thus regulatory interactions, and these changes in turn affect mutation rates mechanistically. Thus, selection exists, but does not act on accidental genetic variation, because the variation is not accidental in the first place; it is influenced by previous generations of selection at the mutation-specific level through preexisting genetic and epigenetic information. Nonrandom, non-Lamarckian genetic variation is a part of selection, and selection is a part of this genetic variation—the two influence each other inherently and are inseparable. Demonstrating these principles, according to the replacement hypothesis, the origination of gene fusion, gene duplication and the predicted RNA editing–based mutations is not due to mere chance unrelated to the biology of the organism, but instead is influenced in a mechanistic, systematic and mutation-specific manner by phenomena carrying meaningful biological information, such as genes being used together, genes being expressed excessively, and certain nucleotides being edited recurrently at the RNA level. In addition, it is not influenced in general directly by the environment but by information that has accumulated in the genome over time—information which is itself not random but reflects the current biological structure and function of the organism, as has accumulated under previous nonaccidental heritable changes and selection.

### Principles of information acquisition are apparent in the nature of mutation

By arguing that mutations are neither random nor Lamarckian, we do not simply mean that mutation rates vary (Hodgkinson and Eyre-Walker 2011; Rahbari et al. 2016; Carlson et al. 2018). Instead, we mean that the causes of mutation are of fundamental importance for how evolution happens.

Consider that the principle whereby pieces of information that are commonly used together become fused is a fundamental principle of learning. According to Hebbian learning, when one neuron repeatedly and persistently participates in causing another neuron to fire, the synaptic connection between these two neurons is strengthened (Hebb 1949). Thus, it has been said that “neurons that fire together wire together” (Löwel and Singer 1992). Likewise, on the macroscale of brain operation, when actions or pieces of information are repeatedly and consistently used together in a certain context, they are fused together or “routinized” into a new action or unit that can be activated or recalled as one—a fundamental principle of cognition and learning called “chunking” (Lindley 1966; Tulving and Craik 2005). According to the used-fused effect, a similar principle of fusing units of information that are repeatedly used together applies to molecular evolution.

Like nonaccidental fusion, nonaccidental duplication has also been considered a key principle of information acquisition (e.g., Newell 1980). According to the replacement hypothesis, gene duplication does not occur randomly with respect to function, but rather elevated transcription–﻿based duplication duplicates genes whose products are more likely to be needed at higher amounts and whose duplication is more likely to lead to functional specialization. Both elevated transcription–based duplication and used-fused mechanisms demonstrate that fundamental principles of information acquisition are evident in the mechanistic nature of mutation.

### A network of information flow

To better understand the consequences of nonaccidental, non-Lamarckian heritable change, let us expand the purview to include the problem of sexual reproduction, called “the queen of problems in evolutionary biology” (Bell 1982). The reconciliation of biometry and Mendelism, which established the basis for the modern theory of evolution (Fisher 1930), encouraged the conceptualization of genes as separate actors, where selection acts on separate contributions of genes to fitness as opposed to complex wholes (Wade and Goodnight 1998; Ewens 2004). This conceptualization both fit with the notion of random mutation, which rose to prominence at the same time (Morgan 1903; Fisher 1930), because genetic change could be treated as a single-gene event that is independent of other genes and is thus accidental,^3^ and created the modern problem of the role of sex in evolution (Livnat 2013): It is often said that sex generates a vast number of different combinations of genes, and because genetic variation is the fuel for natural selection, these combinations facilitate adaptive evolution. However, what is obtained by subjecting so many different complex combinations of genes to the test of selection, when just as sex puts them together, it also breaks them down in the next generation? The intuition that the combinations are important has been incomplete.

**Fig. 2:**
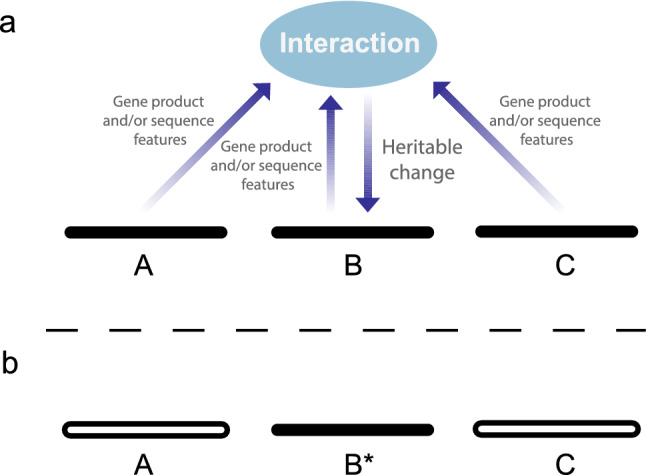
Heritable change puts together information from multiple loci (modified from Livnat, 2013). **a** Without the downward arrow, the figure merely summarizes in schematic form an essential, well-known part of the molecular and cellular biology textbook: genes interact to affect classical traits, like the eye or the ear. However, evidence suggests that there is a heritable change arrow too: that genes interact in affecting heritable change, both genetic and epigenetic. It follows that information is transmitted from the multiple interacting alleles at different loci into the locus being changed by mutation. This generates from the combination of interacting alleles a new heritable piece of information, such as a new mutation at locus B, denoted B*. This new allele is an elementary unit to the sexual shuffling of the genes—it is not in itself broken down by sexual recombination. Therefore, even if the alleles denoted by black lines at loci A, B and C separate due to meiosis (**b**), the transient, complex whole they once constituted does have an effect on future generations through the heritable change that was derived from it. This information is transmitted precisely in accord with the fitness of the organism as a complex whole because transmission depends on survival and reproduction. For the sake of simplicity, only three loci are presented, though in reality many more could affect the interaction

However, according to IBE, even though the combinations themselves disappear, system-level information is transmitted from them to future generations through the heritable changes that are derived from them (Fig. 2). This enables a framework where the basic elements of evolution interact: sexual recombination generates a vast number of different combinations of genes; selection acts on these combinations as complex wholes; and heritable changes that are influenced by complex information in the genome transmit information from these combinations to future generations (Livnat 2013). This information is transmitted precisely in accord with the fitness of the organism as a complex whole, and no Lamarckian transmission of information is involved (Fig. 2). Thus, fundamental open problems hitherto considered separate—What is the role of sex in evolution? How can selection act effectively on individuals as complex wholes? and, What is the fundamental nature of mutation?—correspond to different aspects of one and the same process.

**Fig. 3:**
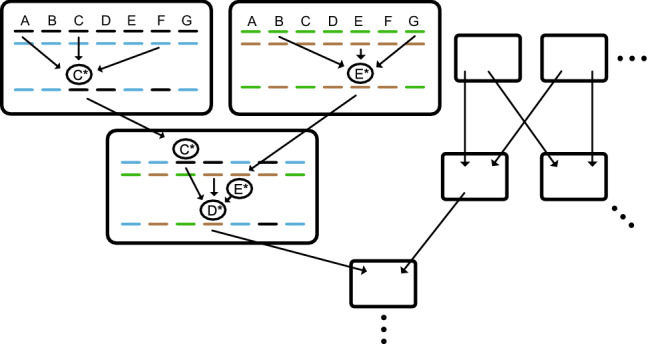
A network of information flow through the genome and generations (from Livnat, 2013). Boxes represent individuals. In each box, the top lines represent the genome of the individual and the bottom lines the genome of a gamete. The three large boxes represent two parents and an offspring. Arrows represent information that affects heritable change. When complex information in the genome, genetic and/or epigenetic, affects the probability of heritable change in a change-specific manner, the outcome of an operation generating heritable change in one generation (e.g., C*) affects the outcomes of heritable change operations in future generations (e.g., D*). Therefore, there is a network of information flow via heritable changes through the genome and generations. Even though the genome of each individual is transient, system-level information is transmitted from it to future generations. As a result, sexual recombination, natural selection and heritable changes that respond to complex information in the genome together combine system-level information from many successful individuals over the generations. For simplicity, only one heritable change per individual is shown, though in reality many exist, considering both genetic and epigenetic changes

Furthermore, because each heritable change that is transmitted becomes a part of the complex information in the genome that affects future heritable changes in a manner specific to each change, there is a network of information flow via heritable changes through the genome and the generations: information flows from many genes into any one gene, and from many ancestors as complex wholes into any one descendant (Fig. 3).

Networks of information flow are of fundamental importance and are key to natural phenomena across the scales of organization, from the living cell to social insects to the human brain. As the neuron, by taking a transient combination of input signals from multiple upstream units, operating on them, and transmitting the outcome to downstream units, ties the network together, so heritable changes that are influenced by complex information in the genome, together with sexual recombination, tie themselves together into a network. However, while networks such as the brain operate within the lifetime of an individual, the evolutionary network of heritable changes spans a much larger scale of space and time. According to IBE, a vast array of biological activity affects the probabilities of heritable changes across the genome. Therefore, the information processing that leads to the evolution of complex adaptations can involve heritable changes in the germline of each of billions of individuals over time, each individual being evaluated as a complex whole by natural selection, where the informational changes in each surviving germ cell are connected to those of other such cells across the generations from past to future. Thus, the evolutionary process is highly parallel and extends over many generations–it is “wide” in space and “deep” in time^4^—enabling system-level information from many successful individuals to come together over the generations via events of nonaccidental, non-Lamarckian heritable change.

### Evolutionary honing in: a gradual evolutionary process leads to discrete mutational change

It is empirically clear that heritable changes influence one another over the generations. It is also empirically clear that this connectedness extends across a spectrum of change types, from common, high-turnover ones such as epigenetic changes, to rare, low-turnover ones such as large chromosomal rearrangement mutations (Livnat 2013, 2017). For example, while gene duplication is mechanistically influenced by the locations of segmental duplications or low copy repeats (SDs/LCRs) (Gu et al. 2008), the generation of SDs/LCRs is influenced by the locations of transposable elements (TEs) (Bailey et al. 2003), the movement of TEs is influenced by sequence characteristics (Graur and Li 2000), sequence characteristics are influenced by point mutations, and point mutations are influenced by epigenetic changes (Fryxell and Moon 2005; Qu et al. 2012). As another example, while the interaction between genes is affected by chromatin states, epigenetic marks, promoters, enhancers and transcription factors, Bolotin et al. (2022) argued that the interaction between two remote genes makes them more likely to undergo a translocation mutation that brings them to the same neighborhood, and that the interaction between neighboring genes makes them more likely to undergo a fusion mutation. Concerning RNA editing, heritable changes that affect the RNA folded structure can expose a new effective A-to-I RNA editing site (Gommans et al. 2009), with further heritable changes contributing to RNA stabilization and honing the target site, leading to increased levels of A-to-I editing that can facilitate an A$\rightarrow$G mutation according to the replacement hypothesis.

This chain of influence across the spectrum of heritable change types can help to explain the otherwise relatively more disruptive, low-turnover changes. According to the replacement hypothesis, the evolutionary process gradually hones in over the long term on genomic regions, specific positions in those regions and specific changes in those positions that are of particular relevance to the currently evolving adaptations. Thus, the accumulation across loci of frequent heritable changes of minor effect under selection leads in the long term to the increase in the rates of rare, large-effect mutations that are more relevant to the current selection pressure and potentially less disruptive compared to other such large-effect mutations that may have occurred if mutation were simply accidental. This connectedness across change types also bears on the general role of epigenetics in evolution: according to IBE, this role is not to enable Lamarckian transmission of information from soma to germline (Jablonka and Lamb 2014; Bonduriansky and Day 2018) but is in being an important part of the spectrum of heritable change on the high-turnover side that meshes with and influences the other, less frequent changes on the intergenerational timescale. Since epigenetic marks often operate en masse (Duret and Galtier 2009), and given their nonaccidental, generally non-Lamarckian nature, they enable a connection between the gradual evolutionary process of their accumulation and the more punctuated DNA mutational changes.

We also add that long-term directedness in mutation origination does not mean that all heritable change is beneficial; on the contrary. Because IBE is not Lamarckian, it relies on selection to evaluate the consequences of mutation; and because it is difficult to change a complex whole without causing harm, the evolved mutational pressure is expected to lead to genomic points of friction between the long-term process of adaptive evolution and the short-term consequences of recurrent genetic disease (Livnat 2013), for which evidence exists (e.g., Inoue and Lupski 2002; Clark et al. 2003; Sharp et al. 2006; Nguyen et al. 2006; Voight et al. 2006; Crespi and Summers 2006; Zhao et al. 2010; Dumas et al. 2007, 2012). Thus, the observed empirical fitness distribution of mutation is in no contradiction to the principles of IBE. On the other side of the coin, relying on selection does not imply that mutation is accidental. According to IBE, the distribution of mutation rates across the genome is continually evolving and carries meaningful biological information, and therefore its particular form at any one time is critical for the evolution of adaptations occurring at that time.

### The ultimate source of heritable novelty

Neo-Darwinism holds that random mutation “invents” a new phenotypic change and natural selection “tests” it (Morgan 1903; Dawkins 1986; Lenski and Mittler 1993). However, according to IBE, phenotypic novelty does not arise from any single mutation: it arises from many mutations that interact with each other at the network level (Livnat 2013, 2017).

In accord with the replacement hypothesis, IBE offers how the mechanistic nature of mutation enables such interactions to begin with: While mutational phenomena bring about local simplification, natural selection ensures that processes are only simplified to the extent that they keep working. This simplification under performance pressure generates from preexisting interactions new elements that have an inherent capacity to come together into novel interactions at the system level. Thus, random mutation is not needed for producing new, heritable information. This information arises at the system level (Livnat 2017).

The connection between local simplification and a global increase in complexity can be illuminated by examples from organismal-level evolution. One such example (for many others, see Livnat 2017) is the evolution, in ground-nesting birds, of the retrieval of an egg that has rolled outside of the nest (Watson and Lashley 1915; Kirkman 1937; Tinbergen 1960). In terns (e.g., *Onychoprion fuscatus*), when the bird notices an egg outside the nest, it stands up and walks to it. However, because it is reluctant to leave the nest while brooding, it sometimes turns around and returns to the nest without having reached the egg. Sometimes, though, it reaches the egg just near enough as to be able to apply the “shifting motion” to it—an ancient, fused sequence of operations normally performed inside the nest and thought to ensure even temperature distribution to the eggs, whereby the bird puts its beak over an egg, rolls it until it is under its breast, and then immediately sits on it to incubate it (Caldwell and Cornwell 1975). Having sat to incubate the egg, it soon notices its nest again, stands up, walks back to the nest, and sits to incubate the eggs there. Soon it notices the outside egg again, walks to it and applies the shifting motion, and so on. Each time it applies the shifting motion, the egg rolls 2–3 inches toward the nest, and, after several such round trips, the egg finds its way back to the nest (Watson and Lashley 1915). However, in the nightjar (*Caprimulgus europaeus*) and other species, the bird walks straight up to the egg, puts its beak over it, and walks backward all the way to the nest, shifting and dragging the egg back in one shot (Kirkman 1937; Tinbergen 1960). Based on various evidence, Tinbergen argued that this single-shot retrieval evolved from a behavior akin to that observed in terns (Tinbergen 1960).

We see that this adaptation originated in a haphazard manner, where different, independently controlled, preexisting actions had been coopted into a novel, emergent interaction, and was subsequently streamlined through simplification and fusion: the visual stimulus originally needed in order to return to the nest was simplified away, and reaching the egg, grabbing it and returning to the nest have fused together into an automatic sequence of operations, forming a new, simple, behavioral module.

No less important, simple, elegant modules were involved in the origination of unstructured egg retrieval in the first place: broodiness draws the bird to egg-like objects, leading it both to the outside egg and back to the nest; broodiness also makes the bird reluctant to leave the nest, so that it stops as soon as it can reach the egg with its beak, which causes the egg to be drawn closer; and the egg only rolls in the direction of the nest because the bird walks in straight lines, so that shifting points backward in the right direction. This and many other examples show that, when elements are both simple and functional, their usefulness tends to extend beyond the original context of their usage, which enables them to come together into unexpected, useful interactions at the system level. Consequently, the egg returns to the nest despite a lack of intentionality. Next, simplification of this novel interaction creates a new trait—egg retrieval by backward walking—which is now available for engaging with other elements in emergent interactions in the future. Thus, novelty does not arise from random, point-wise change. It arises from emergent interactions and is then honed—simplified and generalized (Livnat 2017). With single-shot egg retrieval, birds can colonize areas where the eggs can be blown by the wind a great distance.

The fact that what is both simple and functional generalizes—basic to processes of information acquisition across realms (Livnat 2017)—helps to explain the phenomenon of cooption. In cooption, an element that serves one function gradually comes to serve another in the course of evolution (Gould and Vrba 1982; Hallgrímsson et al. 2012; Müller and Wagner 1991; Graur and Li 2000). Cooption is ubiquitous in evolution and key to the evolution of adaptations, yet little has been said about its fundamental causes. The evolution literature treats cooption as though it just so happens (Williams 1966; Gould and Vrba 1982; Gould 2002). However, according to IBE, both mutation and cooption have important causes, and their causes are interrelated: mechanisms of heritable change, such as the replacement mechanisms above, enable simplification, and the combined pressures of simplification and fit generate elements that have an inherent capacity to come together into novel, useful interactions at the system level (Livnat 2017).

This addresses a fundamental question: Had the organism already known how to produce a beneficial heritable change in direct response to a specific environmental pressure, as in Lamarckism, this would have only begged the question of how it had evolved that particular knowledge to begin with. On the other hand, if it has no a priori knowledge of the specific evolutionary solution required, how could the solution be reached, except by being stumbled upon by accident? This question begets the traditional dichotomy of accidental mutation vs. Lamarckism (Futuyma 1998; Merlin 2010; Razeto-Barry and Vecchi 2017). However, according to IBE, novelty is not simply stumbled upon by chance. Instead, there is a way to “put work in, and get novelty out”: simplification under performance pressure expectedly leads to unexpected, useful interactions at the system level. In other words, once we focus on the system level, we see that random mutation is not required for producing new heritable information. While each heritable change is driven mechanistically by its own proximate causes, novelty arises from emergent interactions between these changes at the system level (Livnat 2017).

From the rm/ns perspective, cases such as the HbS mutation one fostered a reductionist view, where all that was needed was for random mutation to generate the A$\rightarrow$T change and then selection has done all the rest. In contrast, we argue that traits arise from interactions of preexisting elements at the system level, and that this principle applies also to malaria resistance and the HbS mutation: the HbS mutation did not arise accidentally and did not initiate a process of adaptation but rather arose from preexisting interactions that resulted from a long-term evolutionary process.

## How to explain the HbS mutation’s de novo origination patterns

Earlier in this paper, we raised the question of how the genome could “know” to increase the rate of the HbS mutation in the gene and in the population where it is of adaptive significance. We then proposed, and demonstrated with examples, that various types of mutations relevant for adaptive evolution under selection could arise mechanistically and directly from previously evolved interactions. Livnat (2013) furthermore hypothesized that the gradual gathering of system-level information from many successful individuals over the generations by the interaction of sexual recombination, nonaccidental mutation and natural selection allows mechanisms of heritable change to converge on the commonality between successful individuals and thus on genetic interactions relevant to the currently evolving adaptations (Livnat 2013). Thus, from the accumulation of heritable changes of minor interactive effects across loci through the generations, heritable changes of major effect follow mechanistically and directly at relevant base positions and genes (Livnat 2013). This view draws a direct and mechanistic link between the evolution of regulation—considered a rapid and flexible process (Carroll 2008; Wagner and Lynch 2010; Jones et al. 2012; Fraser 2013; Gokhman et al. 2021; Agoglia et al. 2021)—and structural mutational changes. Specifically in the case of malaria, many regulatory and coding regions affect directly and indirectly the within-host environment encountered by the parasite. Thus, we hypothesize in broad outline that small phenotypic variation in the ability to resist malaria that was due to many different genetic causes and had a complex genetic basis was initially present in the population as a result of other evolutionary processes, in line with the ubiquity of cooption; and that from this variation, a gradual evolutionary process based on the principles of IBE honed in on changes in particularly relevant positions in the hemoglobin and other relevant genes, including the HbS mutation in sub-Saharan Africans (Melamed et al. 2022).

While the general process responsible has been outlined above, how it may apply in detail to the HbS mutation remains to be uncovered. We provide below some observations to help propel the quest for the missing detail.

Focusing on hemoglobin genes in saker falcons, Pan et al. (2018) observed a correlation between the expression levels of genes and their mutation rates in blood samples, along with characteristics of transcription-associated mutations (TAM). These single-strand mutations become fixed as de novo mutations in the daughter cells after DNA replication during erythropoiesis, giving rise to numerous mutation variants in the soma and to an increased diversity of alternatively spliced mRNA variants due to de novo splice sites (Pan et al. 2018). Pan et al. argued that this effect is strong in the hemoglobin genes because they are highly expressed and even stronger in a falcon population living at a high altitude, where there is increased oxygen demand. They also found that more than 80% of the hemoglobin mutations were to T, and that the most common mutation type was A$\rightarrow$T on the coding strand (Pan et al. 2018). These observations are of interest given that the HbS mutation is an A$\rightarrow$T one and that it was the point mutation of highest de novo rate in the sub-Saharan African hemoglobin subunit beta (*HBB*) gene in the HbS de novo mutation study (Melamed et al. 2022). These observations raise the possibility that also in humans, a greater amount of relevant RNA diversity may have evolved in African populations that have been subject to intense malarial selection pressures compared to northern European populations, and that the HbS mutation arises as a type of replacement following evolved complex genetic interactions that have not yet been charted.

Importantly, mutation rates in Pan et al.’s study were more complex than simply being based on transcription. They were lower in gene bodies in methylated regions, were highly nonuniform across positions, and appeared particularly high at de novo splice sites based on the observation of many spliced variants among small cDNA samples (Pan et al. 2018). These observations appear to be more consistent with the concept of regulatory processes influencing mutation-specific origination rates than with the errant, scattered genetic change implied by the concept of random mutation. Thus, we hypothesize in broad outline that a complex set of pre-evolved phenomena may be increasing the rates of specific hemoglobin mutations in specific human populations. A DNA hairpin structure due to a local palindrome at the site of the HbS mutation (Alvarez-Dominguez et al. 2013) may also be involved in the specificity of the mutations generated in the *HBB* site, although nonlocal information must exist that interacts with these features (Livnat 2013) in order to explain the observed difference in the HbS mutation rate between the African and northern European populations (Melamed et al. 2022).

As in the previous cases of replacement discussed, mutational replacement involving TAM need not involve direct transmission of information from soma to germline. Gradual increases in the rates of specific genetic changes due to complex interactions in the soma, accepted by selection over the generations, may come together with smaller gradual increases of the corresponding mutation-specific rates in the germline due to partly overlapping mutational mechanisms, leading to replacement and simplification. Consistently, Park et al. (2012) noted a significant correspondence between germline TAMs and somatic gene expression. Again as in the previous cases of replacement, the potential of TAM-involved replacement for facilitating evolution can be seen: transcription beyond the norm for a certain gene may be correlated with instability following a recent environmental pressure that the organism has not yet evolved to fully counter, and further changes in such a gene are more likely to be relevant for the evolution of adaptation. Additional factors may focus mutational change on particular mutations and positions within such a gene.

At some point in the evolutionary past, however, there may have been no 20A$\rightarrow$T somatic DNA or a 20A-to-U RNA change, even if such a change later appeared and was replaced by the HbS mutation. Therefore, we note that cooption both increases the range of possibilities for replacement and is needed for evolutionary novelty: the ultimate origins of a 20A$\rightarrow$T change could be in cooption of a different but related change. Pre- and post-cooption traits are generally related in their biological meaning (Livnat 2017), enabling a gradual evolutionary process where evolved interactions that occur repeatedly over the generations lead directly and mechanistically to heritable changes that may carry some but not necessarily all of the meaning that the previous interactions had and that may take the latter’s place.

Thus, while the molecular details of what causes the de novo HbS mutation patterns are purely speculative, of first and foremost importance in this regard is the system-level view of mutation origination that is consistent with these patterns. We argue that what the details of the HbS mutation mechanism are is not the final question: there is no “homunculus” in the genome that directs different mutations adaptively, and no single mutational mechanism that gives rise to all mutations. According to the system-level view of mutation origination proposed here, even just the HbS mutation alone could have arisen after a series of heritable changes, each of which originated due to its own proximal complex causes. There may be an enormous array of interrelated mutational phenomena, themselves continually evolving, of which only a small amount may be known at the present time.

## Consequences of the system-level view of mutation origination

The explanatory power of the replacement hypothesis affects diverse topics, such as directed mutation, the evolution of genome organization, parallel evolution and the contribution of transposable elements to the evolution of gene regulatory networks.

### Long-term directed mutational responses to specific environmental pressures are possible

Empirical data on the nature of mutation have been collected and interpreted so far through the lens of the traditional dichotomy between random mutation and Lamarckism (Futuyma 1998; Merlin 2010; Razeto-Barry and Vecchi 2017). Therefore, the lack of evidence for Lamarckian mutation in Luria and Delbrück’s (1943) fluctuation test has been taken as an empirical proof of the random, accidental mutation concept (Futuyma 1998; Lenski and Mittler 1993). However, according to IBE, this dichotomy is too limiting.

According to Lamarckism, a direct phenotypic response to an environmental change that occurs within the lifetime of the organism can induce beneficial heritable change, indeed in a manner that circumvents natural selection. An example would be a unicellular organism sensing its environment and cognizantly producing beneficial heritable change in response to that sensing (Shapiro 2011). In contrast, IBE holds that differential survival and reproduction provides the feedback on the fit between the organism and its environment. Importantly, however, differential survival and reproduction itself is based on heritable changes whose origination was influenced by complex information in the genome. Because this information is accumulated in the genome over the generations, long-term directed mutational responses to specific environmental pressures are possible (Livnat 2013). That is, while genetic and epigenetic heritable changes occur at each generation, large-effect, easily observable ones that are particularly relevant to resisting the new environmental pressure often may take multiple generations to arise via evolutionary honing in and mutational replacement (although sometimes they may appear immediately and give the appearance of Lamarckism;^5^ Hull et al. 2017). Therefore, whereas previous studies looked for an immediate, directed mutational response to an environmental challenge (Luria and Delbrück 1943; Cairns et al. 1988), IBE led us to compare the HbS mutation rate between populations that had been subject to different malarial selection pressures for an estimated 10,000 years (Kwiatkowski 2005). Consistently, results showed that the rate was higher both in the gene (comparing 20A$\rightarrow$T in *HBB* and *HBD*) and in the population where HbS is of adaptive significance (Melamed et al. 2022). We expect that further studies of mutation rates in nature will be able to generalize these results to other genes and organisms, and that future experimental evolution studies designed according to the principles of IBE will be able to further support these observations using various model organisms and target genes.

### Mutational replacement and the evolution of genome organization

From the perspective of rm/ns, Gilbert’s famous hypothesis of exon shuffling (Gilbert 1978) can be taken to mean that the existence of introns reduces the chance that a random mutation will translocate one exon into another and disrupt the latter, and increases the chance that it will fall between other exons and form a new and useful combination of exons there. However, according to the replacement hypothesis, exon shuffling is not driven by accidental mutation but by mutational replacement mechanisms: an interaction of exons from afar can lead mechanistically and directly to a translocation which turns those exons into neighbors, and an interaction between neighboring exons can lead mechanistically and directly to a DNA fusion mutation (Livnat 2017; Bolotin et al. 2022). Furthermore, trans-splicing can lead directly to cis-splicing, and cis-splicing to fusion (Livnat 2017; Bolotin et al. 2022).

Replacement-based exon shuffling provides a simpler explanation than rm/ns to cases where the same exons are trans-spliced in one species or population and cis-spliced in another (Fischer et al. 2008; Labrador and Corces 2003; Shao et al. 2012; Kong et al. 2015), or where some functions are achieved by multiple single-module proteins in one taxon but by a single multi-module protein in another (Henikoff et al. 1997; Graur and Li 2000). In addition, recent empirical evidence for the used-fused effect (Bolotin et al. 2022) supports replacement-based exon shuffling. Thus, we argue that exon shuffling and alternative splicing are directly connected via mutational replacement mechanisms. Because exon shuffling is a phenomenon in evolutionary time and alternative splicing is a phenomenon in developmental time, this offers a direct link between evolution and development via mutational mechanisms. In addition, we argue that the evolution of genome organization can be driven by mutational replacement mechanisms (Bolotin et al. 2022) as opposed to random mutation and random genetic drift (cf. Lynch 2007).

### Mutational replacement and the repeatability of evolution

It follows from the replacement hypothesis that parallel adaptive evolution may be not only due to similarities in selection pressures and phenotypic constraints between genetically related species (Blount et al. 2018) but also due to similarities in mutational tendencies: if mutations respond to complex information in the genome, the more genetically related two biological entities are, the more similar their mutational tendencies should be (Livnat 2013, 2017). This principle, which may be called “genetic relatedness in mutational tendencies,” is consistent with the observation that similar malaria-related mutations tend to appear repeatedly on different genetic backgrounds within a human population, whereas different such within-population-repeating mutations appear in different human populations (Livnat 2013). This principle has been empirically supported by the population-level differences in the HbS mutation and other mutation-specific rates demonstrated by the HbS study (Melamed et al. 2022). Further consistent with it, while studying the used-fused effect, Bolotin et al. found evidence for extensive parallelism in gene fusion mutations (Bolotin et al. 2022). Thus, under IBE, parallelism in evolution may be far more common than previously recognized. It follows that the sharing of a mutation by a monophyletic group does not immediately imply that the common ancestor had the mutation, because there is a possibility that the common ancestor had the genetic background on which that mutation was more likely to arise later multiple times.

### Transposable elements and the evolution of gene regulatory networks

Despite the fact that transposable elements (TEs) have been perceived as “selfish elements” at the DNA level (Dawkins 1976; Doolittle and Sapienza 1980), recent evidence has clarified that their contribution to adaptive evolution is immense (Lynch et al. 2011; Emera and Wagner 2012a; Fedoroff 2012; Chuong et al. 2013; Ellison and Bachtrog 2013; Notwell et al. 2015; Chuong et al. 2016). This evidence supports the initial proposal that, by inserting multiple copies of itself and its transcription factor binding sites at different loci, one TE can become a master coordinator of multiple genes (Britten and Davidson 1969; Lynch et al. 2011, 2015). Using the replacement hypothesis framework, we offer an explanation for how and why such insertions occur: In a gradual evolutionary process, a set of genes, each previously active in other contexts, first come to interact in an emergent, novel network fulfilling a novel, complex function (Lynch et al. 2015). As a result, the chromatin at these cooperating loci is open at the same time—according to the replacement hypothesis, also in the germline (Livnat 2013). A TE active at the same time in the germline could then insert itself into these open loci. A positive feedback loop could furthermore arise, where this TE’s activity becomes more and more focused at the relevant time window and locations in the nucleus as it inserts additional copies of itself into the interacting loci.

The process as a whole is one of mutational replacement and simplification: At the early stage of cooption of genes into a novel network, where a complex interaction emerges at the system level in a haphazard manner, different genes are activated by different pre-evolved arms of regulation, consistent with the nature of early complex adaptations in general (Livnat 2017). By contributing the same set of ready-made or cryptic regulatory elements (Emera and Wagner 2012a, b), the TE then comes to partly or fully replace the previously distinct arms of gene regulation with one control. The result is simplification of network regulation through mutational replacement.

Thus, rather than the contribution of TEs being either to the genesis of novel gene regulatory networks (GRNs) or to the turnover of regulatory elements within an existing GRN (Lynch et al. 2015), these may be not two mutually exclusive possibilities but different aspects of the same process: the subjecting of a previously disorganized set of coopted cooperating genes, each activated by its own preexisting arm of regulation, to a common control is a part of an evolutionary process of simplification and routinization, exemplifying the IBE principle that a complex adaptation emerges in a fuzzy, disorganized state and is then crystallized as a whole into a clockwork-like state through a gradual evolutionary process of simplification under performance pressure (Livnat 2017).

### Mutational replacement, genetic assimilation and the Extended Evolutionary Synthesis debate

In a recent debate on the nature of evolution, geneticists argued that only random mutation, natural selection, random genetic drift, recombination and gene flow are the basic processes that produce evolutionary change (Wray et al. 2014). Opposing this, supporters of the Extended Evolutionary Synthesis (EES) contended that organismal-level considerations are also important, in the sense described by the “phenotype first” view: the phenotype first responds to environmental challenge, and then genetic change follows, which allows a coordinated and functional (Laland et al. 2015) response to the environment that meaningfully directs further evolutionary change (see also, e.g., Waddington 1942; Schmalhausen 1949; West-Eberhard 2003).

However, for this proposal to work, and not by Lamarckism, it must rely on some form of accommodation or genetic assimilation to allow the phenotype-first response to become a heritable, constitutive part of the organism that can be built upon as a part of long-term adaptive evolution, a necessity that seems to be recognized by both sides of the debate (Laland et al. 2015; Wray et al. 2014). However, so far, mechanisms of accommodation or genetic assimilation relied implicitly or explicitly on random mutation. For example, West-Eberhard invoked selection on modifier alleles without deviating from traditional principles (West-Eberhard 2003), Waddington and Schmalhausen invoked canalization or stabilizing selection (Waddington 1942; Schmalhausen 1949), and Stern’s classic assimilation model invoked selection on alleles of additive effects with a linear threshold term, ignoring mutation origination entirely (Stern 1958; Falconer 1960). Given this implicit or explicit reliance on rm/ns regarding how genetic change can follow phenotypic change, one can understand the critics’ difficulty in seeing how the EES goes beyond the traditional view (Wray et al. 2014): Because random mutation bears no logical connection to the preexisting structure and function of the organism, in some sense, in order for a response to be first phenotypic and later genetic, random mutation, natural selection, etc., must “reinvent” the phenotypic response in order to provide it with a stable genetic basis, to the degree that the phenotypic response was not genetically determined to begin with. If so, questions arise such as whether all that the phenotype-first response adds is an ability to withstand an environmental pressure that provides rm/ns extra time to reinvent, thus serving as a secondary effect that facilitates evolution in accord with traditional principles (Wray et al. 2014), known since Baldwin (Baldwin 1896).

However, here the replacement hypothesis offers a very different solution. Quite the opposite of random mutation, which bears no relation to the preexisting structure and function of the organism, IBE argues that mutational mechanisms lead directly from the preexisting system-level phenomena to the specific mutations that replace them. Therefore, there is no need to wait for rm/ns to reinvent the phenotypic response: genetic change follows directly and mechanistically from emergent complex change. Innovation originates at the system level without being reducible to rm/ns (c.f. Wray et al. 2014).

Note, however, that this is not a phenotype-first view, as it does not argue that the phenotype necessarily changes first and the genotype second. Rather, the evolutionary response always has a heritable basis, and thus phenotype and genotype change evolutionarily together. However, through gradual honing in on regions of particular relevance for the evolving adaptations, originally diffused, complex heritable effects are ultimately replaced with large-effect, local heritable changes. In that sense, it may seem as though genetic change follows phenotypic change, but the basic principle is that the organism evolves as a complex whole.

This now offers to address two questions arising from the EES’s argument. Consider the plant *Sagittaria sagittifolia*, which has both aerial and aquatic leaf forms (Schmalhausen 1949). As Schmalhausen noted, plants can fix a facultative response in the corresponding stable environment (Schmalhausen 1949), allowing *S. sagittifolia* a purely aquatic or a purely terrestrial existence. Thus, the phenotype first responds through developmental plasticity, and later in evolution constitutively loses the leaves that it no longer requires. The two questions are as follows: First, what is the mechanism by which genetic change follows phenotypic change? If it is rm/ns, then rm/ns is still at the basis of evolutionary theory, and no fundamental conceptual change is required. Second, why is the phenotype able to produce both aerial and aquatic leaves in the first place? It is the origin of novelties that needs to be addressed in the first place.

According to IBE, both questions are part and parcel of the same problem and can be addressed in one: There is no reinvention of a phenotypic response by random mutation. Emergent, complex genetic interactions lead through mutational mechanisms to further genetic change that directly replaces them. Thus, innovation arises at the system level and cannot be reduced to traditional principles such as random mutation. In addition, the system level is innovative because of the nature of mutation—because of the consequences of the combined pressures of mutational simplification and selection.

## Summary and outlook

According to the replacement hypothesis, mutations can follow directly and mechanistically from previously evolved interactions between genes, replacing and simplifying those interactions with heritable change. Heritable changes thus produced form emergent interactions with other such changes at the system level, obviating the need for random mutation in accounting for the origination of new heritable information. Thus, we argued that the HbS mutation did not originate at random and did not begin a process of adaptation by natural selection, but rather a long-term evolutionary process preceded it that has led to its increased rate of origination in the gene and population where it is of adaptive significance.

The replacement hypothesis is a part of the theory of interaction-based evolution (IBE), according to which evolution is driven by the interaction of two forces: an external force of differential survival and reproduction, and an internal force of nonaccidental, non-Lamarckian heritable change. Each heritable change has its own specific origination probability, which depends on complex genetic and epigenetic information accumulated in the genome. This information in turn has come from previous heritable changes and previous selection pressures. Both the external and internal forces are continually updated through the generations: as the organism changes, so does the selection pressure gradually change; and as the genome changes, so do the rates of heritable changes gradually change across the genome. Thus, these two forces continually interact as they coevolve, and their interaction drives evolution. Furthermore, since heritable changes in one generation affect the origination rates of changes in later generations, a network of information flow exists through the genome and the generations. Thus, natural selection, sexual recombination and heritable changes that respond to complex information in the genome enable system-level information from many individuals that have succeeded in survival and reproduction to be combined over the generations.

Many topics are informed by this view, including directed mutation, evolutionary parallelism, genome organization evolution and more, enabling a variety of concrete statements and predictions: (a) Because mutation rates respond in a mutation-specific manner to complex information that accumulates in the genome through the generations, long-term directed mutational responses to specific environmental pressures are possible. (b) These responses are not always beneficial; the gradual process of honing in on regions of importance for adaptive evolution can lead to points of friction between adaptive evolution and recurrent genetic disease. (c) Mutational replacement forms a direct mechanistic link between gradual evolution of regulation and structural mutational changes. (d) The evolution of genome organization is largely driven by mutational mechanisms rather than random mutation and random genetic drift. (e) The distribution of mutation rates across the genome is continually evolving and carries meaningful biological information, and its particular form at any one time is critical for the evolution of adaptations occurring at that time. (f) Genetic relatedness in mutational tendencies exists and contributes to parallelism in evolution (g) Fundamental principles of learning are evident in the nature of mutation. (h) Heritable changes are tied together in a network of influences across the generations and across heritable change types. (i) The basic elements of evolution—selection, recombination and mutation—interact. (j) Mutational replacement mechanisms and natural selection constitute a combined pressure of simplification and fit that generates cooptable elements. (k) A special role for epigenetics in evolution may be not in enabling Lamarckian transmission from soma to germline but in being a frequently occurring type of nonaccidental yet non-Lamarckian heritable change, meshing with and affecting the origination of less frequently occurring heritable changes. (l) TEs tie together gene regulatory networks in a manner that may be explained by the replacement hypothesis. (m) The causes and consequences of mutation are related via mutational mechanisms. Because this connection is due to an overlap between mechanisms operating in developmental time and mechanisms operating in the germ cells, it also forms a connection between evolution and development. (n) Mutational replacement constitutes a form of “genetic assimilation” that is based on nonaccidental, non-Lamarckian mutation. This endogenization by mutation has been a missing piece in the EES debate.

According to this view, a vast landscape of mutational phenomena remains to be studied that is essential for our understanding of evolution. We expect that future natural studies will generalize the HbS results to other genes and organisms and that future experimental studies will be able to further support the HbS observations using various model organisms and target genes by designing platforms for measuring de novo mutation rates at the single-mutation resolution under multigenerational artificial selection pressures and the conditions for long-term adaptive evolution specified by IBE.

## Footnotes

1 Indications exist that the DNA mutation rate due to RNA editing enzymes, though lower than the RNA editing rate, is still much higher than the human genome-wide average mutation rate (Saraconi et al. 2014; Tasakis et al. 2020).

2 Hereafter, we will denote by rm/ns the view that evolution happens by random mutation and natural selection, including the subsidiary effects of random genetic drift, sexual recombination and migration (Futuyma 1998).

3 The notion of random mutation is not Darwin’s own (cf. Darwin 1859, Ch. 5).

4 By using the terms “width” and “depth” from the mathematical study of networks, we do not imply that evolution is an algorithm, though it is capable of creating natural algorithms (Navlakha and Bar-Joseph 2011; Chazelle 2015; Straszak and Vishnoi 2016). We rather view it as a natural system, where the similarities and differences between heritable changes and computational events remain to be elucidated.

5 Sometimes, long-term acting mechanisms can also lead to an immediate response, but when such responses are interpreted from a Lamarckian perspective, they lose their generality (Livnat 2017, p. 178), as in the CUP1 case discussed above.
